# The role of HLA genetic variants in COVID‐19 susceptibility, severity, and mortality: A global review

**DOI:** 10.1002/jcla.25005

**Published:** 2024-01-22

**Authors:** Taraneh Hoseinnezhad, Nasrin Soltani, Sarina Ziarati, Emad Behboudi, Mohammad Javad Mousavi

**Affiliations:** ^1^ Student Research Committee Bushehr University of Medical Sciences Bushehr Iran; ^2^ Department of Hematology, School of Para‐Medicine Bushehr University of Medical Sciences Bushehr Iran; ^3^ Department of Basic Medical Sciences Khoy University of Medical Sciences Khoy Iran

**Keywords:** COVID‐19, disease outcomes, ethnicity, genetic variation, HLA alleles, SARS‐CoV‐2, severity, susceptibility

## Abstract

**Background:**

The COVID‐19 pandemic has had a profound global impact, with variations in susceptibility, severity, and mortality rates across different regions. While many factors can contribute to the spread and impact of the disease, specifically human leukocyte antigen (HLA) genetic variants have emerged as potential contributors to COVID‐19 outcomes.

**Methods:**

In this comprehensive narrative review, we conducted a thorough literature search to identify relevant studies investigating the association between HLA genetic variants and COVID‐19 outcomes. Additionally, we analyzed allelic frequency data from diverse populations to assess differences in COVID‐19 incidence and severity.

**Results:**

Our review provides insights into the immunological mechanisms involving HLA‐mediated responses to COVID‐19 and highlights potential research directions and therapeutic interventions. We found evidence suggesting that certain HLA alleles, such as HLA‐A02, may confer a lower risk of COVID‐19, while others, like HLA‐C04, may increase the risk of severe symptoms and mortality. Furthermore, our analysis of allele frequency distributions revealed significant variations among different populations.

**Conclusion:**

Considering host genetic variations, particularly HLA genetic variants, is crucial for understanding COVID‐19 susceptibility and severity. These findings have implications for personalized treatment and interventions based on an individual's genetic profile. However, further research is needed to unravel the precise mechanisms underlying the observed associations and explore the potential for targeted therapies or preventive measures based on HLA genetic variants.

## Graphical abstract

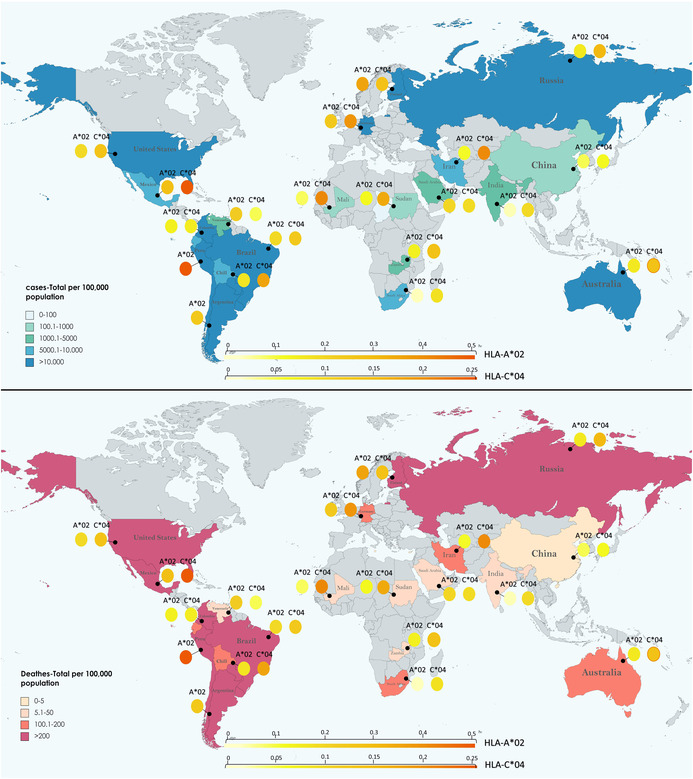

## 1 HLA, IMMUNE RESPONSE, AND SUSCEPTIBILITY TO COVID‐19

### 1.1 An introduction to HLA and immune response

The coronavirus disease 2019 (COVID‐19) pandemic has affected millions of people worldwide, with varying degrees of susceptibility, severity, and mortality rates across different countries and regions. While many factors can contribute to the spread and impact of the disease, recent research has identified a potential role for host genetic variation, specifically human leukocyte antigen (HLA) genetic variants. The HLAs help the immune system recognize foreign substances that could trigger an immune response.^1^ This system was discovered through experiments involving antigen–antibody reactions and the study of genes, leading to the identification of a large gene cluster that encodes proteins on the surface of cells, facilitating immune function.^2^ The HLA genes are known for their high degree of polymorphism, meaning they exhibit variation across a wide chromosomal region.^3^, ^4^ There are three classes of HLA molecules, Class I (HLA‐A, HLA‐B, and HLA‐C), Class II (HLA‐DP, HLA‐DQ, and HLA‐DR), and Class III, which encode proteins involved in cell functions like motility and signal transduction, as well as some complement cascade fragments.^3^ Class I molecules present intracellular peptides to CD8+ T cells, while Class II molecules present extracellular peptides to CD4+ T cells. These distinct mechanisms enable the immune system to distinguish between different types of antigens.^5^, ^6^ Overall, the functioning of the HLA also known as major histocompatibility complex (MHC) system plays a critical role in immunity and is highly regulated and complex.^7^

### 1.2 Role of HLA in susceptibility, severity, and the progression of COVID‐19

Viruses cannot replicate independently; they rely on living cells, and their enzymes orchestrate the production of viral substances.^8^ The glycoproteins of SARS‐CoV‐2, emerging from its surface, exhibit a specific affinity for the gastrointestinal and respiratory tracts by binding to ACE2 and CD147 receptors. The enzyme TMPRSS2 in the cell facilitates the virus's entry, and the severity of symptoms increases with the involvement of more organs.^9^, ^10^ Due to its transmissibility, individuals must avoid being present in public. This is the way World Health Organization (WHO) controlled the SARS and MERS breakouts.^11^, ^12^ Coronaviruses, classified into four groups, can cause a spectrum of illnesses from mild to severe. Attention must be given to the diverse dispersion of these viruses in different countries based on their climates, and their genetic makeup can easily evolve through various mutations.^13^, ^14^ White blood cells (WBCs) play a pivotal role in immunity by circulating through the blood, lymph, lymph nodes, and spleen, encountering antigens in the body. When a virus enters a host cell, it faces both innate and acquired immunity. The collaboration of B cells and T cells works to prevent the virus from spreading through both humoral and cellular immunity. Secondary hemophagocytic lymphohistiocytosis (SHLH) can occur due to an increase in various immune components, including cytokines, and elevated levels of these particles in the body correlate with the severity of infection.^15^ When viruses pose a community risk, the interplay between their pathogenesis and the polymorphism of HLA alleles becomes significant. The more encounters a virus has with the body, the stronger the immune system's reaction, potentially resulting in excessive cytokine production and cell damage.^16^ As polymorphism is associated with peptide sequences, different features will appear in the proteins. The attachment of various host‐viral peptides is compared in epidemiologic studies. The relation between the acuteness of the illness and the class II HLA‐DR zone is high. Hammer et al. discovered that every single person conveying the locus DRB1*15:01 shows noticeable levels of anti‐influenza A IgG.^17^ Later studies on 48 amino acid chains of HCoVs, and the attachment to different HLA alleles, showed that HLA‐A*02:02, HLA‐B*15:03, and HLA‐C*12:03 had high affinities; but among these three, HLA‐B*15:03 had significantly the highest of all. If these alleles express poorly, the mortality to HCoVs might increase and these people must be prioritized for vaccination, since they have less antibody responses against SARS‐CoV‐2 spike protein and the RBD after vaccination.^18^, ^19^

### 1.3 Distribution of HLA haplotypes in the worldwide and susceptibility to COVID‐19

The HLA is a highly polymorphic system that exhibits significant regional variations. Due to the high polymorphism of the HLA molecule and its ethnic dependency, data on HLA allele frequencies can vary widely between populations and sometimes yield conflicting results.^20^, ^21^ For example, an allele that confers protection in one population may be associated with severe disease in another population. It is so difficult to confirm obtained results from every study in comparison to the others because of high polymorphism in the HLA molecule and being ethnicity‐related.^22^ HLA typing is a costly procedure that is more commonly available in developed countries, leading to limited information on HLA allele frequencies in poor or developing nations.^23^ For calculating the frequency of every HLA haplotype, the ratio of dividing number of alleles for each HLA locus is used.^22^ The peptides play a crucial role in the selectivity, diversity, amplitude, and efficiency in both types of immunity responses.^16^ New bioinformatics tools have been developed to predict the affinity between peptides and HLA molecules, facilitating the design of peptide‐based vaccines against viruses such as HIV‐1, Ebola, and SARS‐CoV‐2.^24^, ^25^ Also, the global allele frequency distributions of the strongest and weakest HLA binders were determined by statistical modeling. The strongest binders were identified based on extremely low IC50 (half maximal inhibitory concentration) values and a high predicted affinity to HLA molecule, which by peptide presentation is associated with elicitation of an appropriate immune response. According to the previous studies on the binding affinities, we categorized HLAs, into three groups: strong binders (IC50 ≤ 50 nM), weak binders (500 nM < IC50 ≤ 5000 nM), and none binders (IC50 ≥ 5000 nM) for Class I. Similarly, for Class II, we classified them as strong binders (IC50 ≤ 50 nM), weak (1000 nM < IC50 ≤ 5000 nM), and none binders (IC50 ≥ 5000 nM). In silico studies have been helpful for assessment of the affinity of all SARS‐CoV‐2 viral peptides to various HLA class I genotypes.^16^ According to the findings, the presentation ability of SARS‐CoV‐2 peptides classifies HLA molecules to various categories of affinity (strong, regular, weak, non‐binder). The frequencies of strongest and weakest HLA binders vary considering the geographical regions.^26^ Each allele which is able to recognize a higher number of viral peptides may induce a stronger T‐cell response.^27^ Barquera et al.^26^ identified the strongest binders for SARS‐CoV‐2 as highly effective molecules with appreciable binding affinity for approximately 1% of the presented viral peptides. The weakest binders for SARS‐CoV‐2, as predicted, exhibit poor binding or no association with 99% of presented viral peptides, making them ineffective at triggering an immune response.

### 1.4 Strong binders of the SARS‐CoV‐2 and frequency distribution

#### 1.4.1 HLA‐A strongest binder (HLA‐A locus)

In comparison with most HLA type I alleles, HLA‐A type sound to be stronger SARS‐CoV‐2 peptide binders. HLA‐A molecules especially HLA‐A*02 lineage which has high distribution in globe could be considered as most representative among the HLA class I binders. Most of powerful binders belong to the A*02 lineage except HLA‐A*02:01 which has global distribution particularly in all native American population, and the rest of them including A*02:02, A*02:03, A*02:06, A*02:12 which are very rare.^26^, ^28^ Among these, HLA‐A*02:06 has been predicted to display strong binding affinity.^22^ Initially, in a study, the presence of A*02:01 showed a positive correlation with susceptibility to COVID‐19 and its mortality. This correlation was attributed to its lower capacity to present SARS‐CoV‐2 antigen.^20^ Conversely in the next studies, A02 was identified as having a protective effect against susceptibility and mortality.^29^

#### 1.4.2 HLA‐B strongest binders

At locus HLA‐B, HLA‐B*15 lineage appears to be better binder and correlated with increased risk of COVID‐19 infection as well as death rate. HLA‐B*15:03 is predicted to be most strong HLA allele with high affinity for peptide binding.^30^ In fact, HLA‐B*15:03 is the most potent HLA‐B binder in every virus that their genomes were identified. The other Strong binders of this lineage include (HLA‐B*15:17, 15:25, and 15:39) and also HLA‐B* 35:10. They have very rare distribution among worldwide populations but HLA‐B*15:03 allele is seen in sub‐Saharan Africa.^26^ Maybe this allele is the reason of low mortality rate in proportion to crowd in this continent because of its effective peptide presentation ability.^31^ HLA‐B*15:25 distribution is observed in Australia, South‐East Asia and with high frequency of 40% in indigenous Taiwanese people. Despite, high affinity of HLA‐A alleles is widespread but the majority of the most potent HLA‐B binders are uncommon in the world.^26^ HLA‐B*35 is generally as a current HLA among South Asian people, is identified with high peptide loading capacity compared to other HLA‐B alleles and which might be associated with an efficient immune response but was founded in mildly infected in the study of Naemi et al.^32^ Also, in this study HLA‐B*51 was detected related to mortality and increased in fatal cases, which after statistical correction *p* value became insignificant. Perhaps with more investigation in a larger sample size, significant association be founded.

#### 1.4.3 HLA‐C

The binding capabilities of HLA‐C alleles are weaker in comparison to other type I of HLA and do not bind peptides with high affinity. Among them, HLA‐C*08:02 is rather top‐ranked strongest binder and its frequency was reported in Brazil, Australia, and China.^33^

#### 1.4.4 HLA‐DQ

Any HLA‐DQ is not recognized as a strong binder.^33^

#### 1.4.5 HLA‐DR

According to statistics and bioinformatics strong binders of HLA‐DR are HLA‐DRB1*01:01, DRB1*10:01, DRB1*01:04, DRB1*11:02, DRB1*13:01, DRB1*13:02. Most of the strong binders of HLA‐DR were diffused globally at low to intermediate frequencies, for instances HLA‐DRB1*01:01 was seen in European. Frequency distribution of HLA‐DRB1*10:01, 13:01 were observed in some European, African, South‐West Asian population.^26^ However, in the most studies role of class I HLA molecules as the first line of defense was bolded but also should not be overlooked that HLA type II molecules play an important role for virus‐derived peptides cross‐presentation process.^32^

### 1.5 Weak binder alleles and frequency distribution

HLA alleles that may not be able to present adequate virus‐derived epitope peptides as a result not to incite a sufficient immune response against SARS‐CoV‐2 infection. It is essential to know that SARS‐CoV‐2 weakest binders' alleles could be regular binders for other viruses.^26^ Note that we must be careful to interpret data obtained from studies with small sample size as the conclusion is not definite for this constraint.^32^

#### 1.5.1 HLA‐A

Since A*25:01, A*25:02, especially A*30:04 bind weakly (Weak binder) characterized by a reduced ability to present antigens or even do not effectively bind and present antigens to T cells (non‐binder) to presented peptides, they are categorized as the weakest HLA‐A alleles. The aforesaid alleles have rare frequency but HLA‐A*30:04 is seen in Sudan and Cameroon among African population.^33^ It has been revealed that African American populations carrying HLA‐A*30:04 probably are more susceptible to COVID‐19 compared to other tested races in the Midwest of the US.^29^

#### 1.5.2 HLA‐B

B*44:06, B*51:07, B*08:03, B*46:01, and B*52:01 are seen in the pinnacle of the weakest binders for the same reason mentioned in HLA‐A weakest binder. Some HLA‐B weakest binders have intermediate to high frequency in various areas for example HLA‐B*46:01 in China and South‐East Asia. Interestingly, several HLA‐B weakest alleles were reported at intermediate to high frequency in different geographical areas in contrast to HLA‐A, whereas weakest binders are observed rarely (e.g., HLA‐B*46:01 in South‐East Asia populations and Chinese people and HLA‐B*52:01 is common in Japanese, Indian, Chinese population).^27^ Individuals expressing HLA‐B*46:01 may be more vulnerable to COVID‐19, corroborating previous results showing HLA‐B*46:01 associations with SARS.^34^ Also, HLA‐B*52:01 haplotypes could potentially act as a predictive marker for the severity of COVID‐19 in the Asia region.^29^

#### 1.5.3 HLA‐C

HLA‐C*01:03, HLA‐C*07:04, HLA‐C*07:11, HLA‐C*18:01, HLA‐C*18:02 show weaker binding properties, and HLA‐C18:01 has modest frequencies in Sub‐Saharan African populations.^29^, ^34^

#### 1.5.4 HLA‐DR

HLA‐DRB1*03:02 and HLA‐DRB1*03:03 display the weakest binding patterns among HLA‐C and HLA‐DRB1*03:02 is only observed in a particular population of Africa.^34^

#### 1.5.5 HLA‐DQ

Dimers of DQA1*01/DQB1*06 were introduced as weakest binders amongst HLA‐DQ alleles.^26^ It is important to note characterization of HLA molecules in the act of strongest or weakest binders of SARS‐CoV‐2 peptides is recognized by computational predictions and must be validated with experiments to measure immunogenicity, thus the results are presumptive up to be proved.^26^ MHC‐I alleles particularly are the main elements of the presentation pathway for viral antigens and have been proved to impart resistance or disease severity for various viral infections. Also, HLA class I alleles are detected more in patients with mild COVID‐19 because of better theoretical capacity for binding to SARS‐CoV‐2 peptides and in mild symptom form of COVID‐19 higher heterozygosity is seen as compared with severe form of the disease.^31^ There are so many resemblances in SARS‐CoV‐2 genome and the other members of *Coronaviradae* family, so it is probable to see similarities in alleles that present these viral peptides. SARS‐CoV‐2 illustrates similarity in genome about 77% to SARS‐CoV, so we can expect to presume there is a partly resemblance in the host immune responses against theme. Most of the researches on HLA alleles which could affect SARS‐CoV prognosis have done in Asia, but most of information from other regions are not available to assess SARS‐CoV‐2 data.^28^ Furthermore, the templates of HLA allele affinity for SARS‐CoV‐2 peptides are not specific to this pathogen and it has many similarities to the other mentioned Coronaviruses, SARS‐CoV and MERS‐CoV in the terms of the peptide binding pattern.^26^ Different HLA molecules have variable degrees of affinity for particular viral peptides found in Coronaviruses such as SARS‐CoV and MERS‐CoV which profoundly affects immunity responses procedures and the ensuing clinical symptoms of the disease.^22^ A significant correlation between the emergence of SARS and HLA‐B* 07:03 and HLA‐DRB1*03:01 were beholded in Chinese people. HLA‐B*13 was observed in ICU‐admitted patients with SARS in Chinese patients. Also, HLA‐B*46:01 was reported to be strongly correlated with SARS severity.^35^ HLA‐B*46:01, HLA‐B*07:03, and HLA‐DRB1*12:02 were related notably to more susceptibility to SARS infection among Taiwanese, Chinese, Vietnamese populations, respectively,^28^ but in a related study on 82 Chinese these association were not seen to occur in a significant number of COVID‐19 patients. HLA B*15:27 was the only marker remained significantly correlate COVID‐19 susceptibility after *p*‐value correction in the analysis performed in the Wang et al. study. Because of the small sample size, the significance of these data must be interpreted with caution. Previous studies shown that some alleles such as HLAB*46:01, because of its low binding affinity were considered to confer susceptibility for SARS‐CoV among Asian people.^16^ As a result who carries HLA‐B*46:01 probably confront more severe clinical outcomes during the illness.^36^ This association was declined in the study by Yung et al. possibly because of various factors effect in T‐cell‐mediated immune responses and this process is not limited only to HLA‐peptide interaction, so maybe any special protection or susceptibility is not due to this allele.^37^, ^38^ The more crucial act of HLA‐I alleles in COVID‐19 can be justified by the more determinative act of these molecules in presentation of viral peptides. HLA class‐II molecules participate in design and expansion of peptide‐based vaccines because of stable and long‐term responses and also cross‐presentation.^32^ The weakest binders are HLA alleles that potentially are unable to present adequate number of virus‐derived peptides, whereas HLA‐B*44, HLA‐C*01 alleles have been associated with inflammatory autoimmune disease too and is stressed to their non‐proficient ability to generate immunological reactions. HLA‐C*01 is the most permissive binder to SARS‐CoV‐2 peptides.^16^

HLA‐A*02 is the most investigated allele which was repeated in 18 studies and illustrates contrary results,^28^ so that for example in some studies HLA‐A*02:01 was relevant to elevated possibility of susceptibility to COVID‐19. Particularly, this allele had a partly lower capability to present SARS‐CoV‐2 peptides.^16^ On the other hand, HLA‐A*02 lineage is considered as strong binder with high affinity for SARS‐CoV‐2 antigens in some studies.^28^ HLA‐C*04:01 is one of the alleles relevant to emerge of severity and worse clinical outcomes in European patients, individuals who carry this allele were required to mechanical ventilation more than others.^39^ HLA C*05:01 was identified by one of the activating KIR (KIR2DS4) and this allele was recognized as a risk factor for death in Italy.^16^

COVID‐19 outbreak between northern and southern Italy has shown unexplained discrepancy, so they have set up a study to investigate the hypothesize for the effect of regional prevalence of HLA class I alleles which may underlie to make different immune response,^40^ and only HLA‐B*44,C*01 were correlated with COVID‐19 spreading in the northern region of Italy. In a study by Sardinian et al, both of the HLA‐A*23:01, HLA‐DRB1*08:01 alleles were remained significant after multiple comparison tests. These mentioned alleles particularly were seen in moderate and critical form of COVID‐19 patients respectively. In addition, three loci haplotype of HLA‐A*30:02, B*14:02, C*08:02 maintained strongly correlated with COVID‐19 severity and susceptibility in Sardinian population. A protective effect in Sardinian people was generated due to HLA‐A*02:05, HLA‐B*58:01, HLA‐C*07:01. HLA‐DRB1*08 which is associated with the highest risk for appearing as severe form of COVID‐19 also, and simultaneously in several autoimmune diseases. It could be claimed manifestation of severe clinical outcomes in COVID‐19 patients who carry DRB1*08:01 allele due to altered regulation of cell‐mediated immune responses.^22^ The HLA‐DRB1*04:01 was found a significantly correlation in both of asymptomatic European patients and Iranian patients with milder disease.^18^, ^41^ In other instances, the alleles were introduced accompanied by serious outcomes of COVID‐19 in Egyptian includes HLA‐B*41, HLA‐B*42, HLA‐C*16, HLA‐C*17. Conversely, HLA‐B is strongly linked with protection against mortality due to COVID‐19, so that presence of this allele increased the probability of survival, up to 1351 folds. In the same study, HLA‐B*15 was introduced as the HLA has the great capacity to present extremely conserved SARS‐CoV‐2 peptides common among different human Coronaviruses. HLA B*15 in addition to COVID‐19 has protective function in other infections. Furthermore, HLAB*15, HLA‐C*07, HLA‐C*12 were protective alleles in Egyptians in the Abdul hafiz et al. study. On the other hand, HLA‐B*41, B*42 were related to emerge severe clinical outcomes of COVID‐19. HLA B*41 is one of the repeatedly HLA‐B alleles in the Egyptian population. Many studies proved the presence of some special HLA alleles in relation with susceptibility, severity, or clearance of disease such as HIV, hepatitis, and tuberculosis. Previous study suggested HLA‐C*16 effects on quick transition of HIV to AIDS in patients.^27^ Also, HLA‐C*16 was correlated with acute COVID‐19 pneumonia in Egyptian study. Furthermore, this allele in Spanish COVID‐19 patients had higher rate compared to control group but after correction of *p* value it was not significant.^27^, ^42^

Nguyen et al. in a study predicted that total numbers of peptides derived from SARS‐CoV‐2 was presented by HLA‐B*15 that was one of the cause to survive in Egyptian people and is considered as a top presenter.^36^ In the present study, Africa was mentioned as a mainland with low mortality rate, maybe high allelic diversity (the presence of many different alleles) was demonstrated in Africa compared to other regions which is the reason of lower occurrence of SARS‐CoV‐2 and its related deaths. Also, HLA‐B*15:03 with excellent ability to present SARS‐CoV‐2 peptides, appears to be recurrent in West Africa and in countries with endemic malaria.^31^ In the study of 82 Chinese patients by Wang et al., HLA‐B*15:27 was found probably be related to COVID‐19 occurrence, even though both of these alleles belong to HLA‐B*15 lineage but differ in 10 nucleotides.^20^ Combination of experimental and predictive studies can give us better insights about effect of different alleles on disease prognosis. Published data about HLA‐B*22 seem to suggest a potential risk indication for SARS‐CoV‐2, and on the other hand, HLA‐B*27 has a possible role in dampening COVID‐19. HLA‐B*27 intercedes a protection effect against HIV and hepatitis C virus (HCV).^16^ There is a Chinese study reported a significant association between the presence of HLA‐C*14:02, HLA‐A*11:01, HLA‐B*51:01 alleles in patients and severity of COVID‐19 and its worse consequences. Particularly HLA‐A*11:01 was associated with severe clinical course of COVID‐19 in two distinct Chinese and Japanese cohorts.^39^ In Asian people, HLA‐A*11:01, HLA‐B*51 can be mentioned as markers make a contribution to the severity of clinical manifestations. The A*24:02:01, B*51:01: 01 alleles were found as susceptibility and severity markers among COVID‐19 patients, but these alleles were elevated in healthy control group compared to H1N1/09 infected individuals. Although between SARS‐CoV‐2 and influenza A H1N1 few alleles were common, an allele may not be a good presenter for all peptides. Vice versa in various populations including Chinese, Indian, United Kingdom, HLA‐A*11 and HLA‐DRB1*10 were considered susceptive in both Influenza and SARS‐CoV‐2.^20^ Note that the strongest HLA binders of SARS‐CoV‐2 peptides are common among most of infectious agents with the same mechanism, and probably, they are selected by natural selection a long time ago.^16^, ^26^ Additionally, variants of SARS‐CoV‐2 may have effects on HLA binding and peptide presentation. Also, an HLA haplotype which may effectively present a wild‐type peptide presents a mutant peptide in a different way. Omicron variant is able to make the greatest number of epitopes predicted to bind strongly to two HLA classes. More than 90% of the peptides of SARS‐CoV‐2 variants are presented by HLA class‐II.^39^

## 2 HLA ALLELES DISTRIBUTION IN COUNTRIES

There have been more than 689 million confirmed cases of COVID‐19 since 2020, and the number of cases varies between countries and even between areas within the same country. According to WHO statistics, the fewest and most cases have been reported in Africa and Europe respectively. On the other hand, most affected people were in the United States of America and India.^43^ These differences can be attributed to a variety of factors including social constructions, cultural identity, and behavioral patterns that are common across different ethnic groups. Additionally, genetic variations have a significant role in the spread of viruses and the severity of diseases.^21^, ^44^ In the last decade, a critical technique known as genome‐wide association study (GWA or GWAS) has emerged to investigate the relationship between genetic backgrounds and various phenotypes. The GWAS, by identifying clusters of related SNPs, or genomic risk loci, in one population with a continuous characteristic, can show a statistically significant genotype–phenotype association with diseases and traits.^3^

Some sizable whole genome association studies have been carried out to examine whether host genetic variation influences the susceptibility and severity of COVID‐19. In the first of these studies, Ellinghaus et al., by analyzing the genomes of 1980 COVID‐19 patients with respiratory failure, identified locus 3p21.3131 (near SLC6A20, LZTFL1, CCR9, FYCO1, CXCR6, and XCR1) and 9q34.2 (near ABO) as genetic susceptibility loci in these patients.^45^

Horowitz et al. reported how ACE2 expression levels affect COVID‐19 risk in the other genome‐wide association study. After replicating eight independent associations with disease risk reported in three previous GWAS, six variants were identified with a significant association, four of which, including MHC, DPP9, and IFNAR2, and a variant in/near LZTFL1, showed a significant relationship with worse complications.^46^

In Pairo‐Castineira et al. and the COVID‐19 HGI study, the variants were revealed in a region of chromosome 6 (the major histocompatibility complex). They were all removed from the list of reported loci due to the difficulty of classification and high levels of estimated effect size vary among the studies included in the analysis.^47^, ^48^ Despite the fact that previous GWAS have shed light on the complications of COVID‐19, except for three studies that revealed strong SNP interaction signals at the HLA region, no association was reported in the other studies.^49^ After all, the requirement to use a substantial correlation value to adjust for the multiple test load created by the large multitude of loci being analyzed simultaneously is a limitation of GWASs. As a result, it can overlook crucial associations.^50^ In addition to these studies, several more research carried out in diverse populations and make it very evident that patient HLA profiles can affect onset and outcomes of COVID‐19.^34^ We compiled in a table the significant findings of numerous of these studies. Ethnic categorization techniques might be helpful in analyzing population differences in terms of genetics and other features,^44^ though the ethnicities were included in these studies and links between HLA polymorphism, COVID‐19, and ethnicity or region have been shown in some studies. We do not aim to make any demographic assumptions for some limitation:

1. Additional factors like mask adherence, provision of healthcare, and intensive care bed rates could impact the incidence or outcome of disease.^51^

2. In several research, a design and test combination that was insufficiently powered to detect potentially interesting effect sizes was caused by a small sample size, and in order to increase the possibility of a thorough mapping of HLA disease correlations, many cases from each community should preferably be pooled in a global context.^52^, ^53^

3. Heterogeneity in study designs. For instance, the primary focus of Langton et al. study was to define between groups with severe and those without symptoms, However, patients who had been admitted to the hospital for a COVID‐19 infection were included in a study of HLA in Italia.^18^ So, a global effort is required to acquire data from various groups of patients in order to conduct more statistically strong studies.^54^

4. The various allele frequencies reported range significantly between areas, so it is possible that risk alleles revealed in previous research have not been significant in other groups.^54^

**FIGURE 1:**
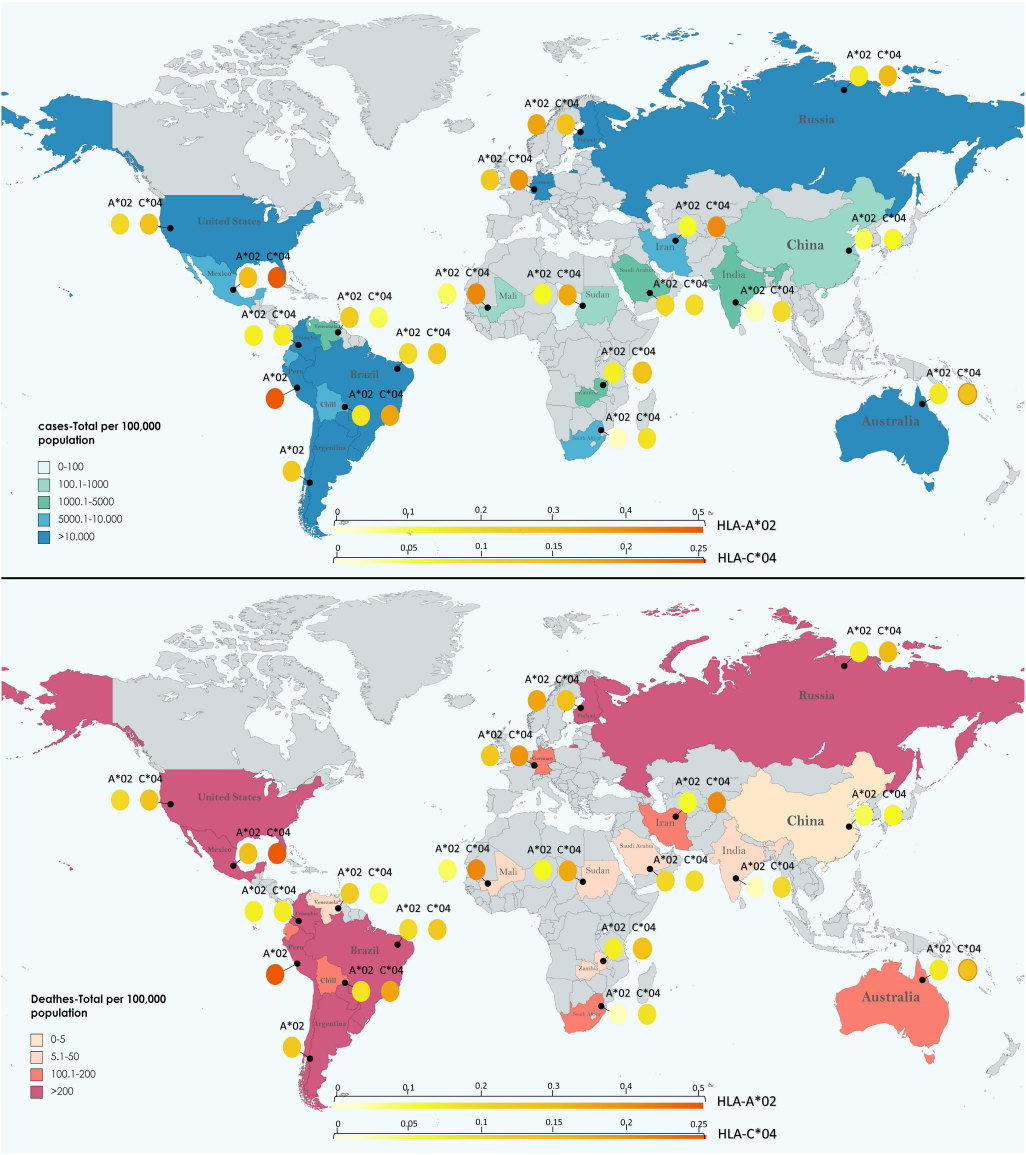
Impact of HLA‐C04 and HLA‐A02 Alleles on COVID‐19 susceptibility and mortality rates. This figure presents a worldwide vision illustrating the relationship between the frequencies of HLA‐C04 and HLA‐A02 alleles and their respective associations with COVID‐19 susceptibility and mortality rates across different regions. The darker shaded areas represent regions with moderate to high frequencies of selected alleles and higher rates of susceptibility and mortality from COVID‐19. However, there are exceptions, such as China, where despite a higher frequency of HLA‐C04, lower incidence and mortality rates are observed. This suggests the influence of factors beyond host genetics, including government policies (e.g., Zero‐COVID‐19 policy), timely implementation of quarantine measures, access to vaccines and treatments, and well‐equipped medical facilities. Similarly, the figure highlights the protective effect of HLA‐A02 allele, which is associated with lower COVID‐19 susceptibility and reduced mortality rates. However, variations in this protective effect are observed, possibly due to the aforementioned contributing factors.

Despite these limitations, and sometimes contradictory results, such as the findings for HLA‐B44 (Table 1), the findings of these research have led to the identification of common alleles in some instances. Three of them demonstrated the preventive benefits of HLA‐A*02 against susceptibility and mortality in a clinical setting with COVID‐19 patients and healthy controls.^22^, ^55^, ^56^, ^57^ Binding affinity of HLA alleles with SARS‐CoV‐2 peptides could be a potential reason for how a HLA allele affects the severity of the disease.^53^ According to an analysis of viral peptide–MHC class‐I binding affinity for SARS‐CoV‐2 peptides, HLA‐A*02 was one of the three representative alleles with the highest predicted capacity. While HLA‐C*04 was indicated as a weak binder.^36^ HLA‐C*04 was the most investigated HLA and positively correlated with SARS‐CoV‐2 susceptibility and severity.^22^, ^52^, ^58^, ^59^, ^60^ HLA‐C*04, which can double the risk of intubation even when present in only one allele, was identified as a potential risk allele in research conducted by J. Weiner et al. This finding was made in an important study that included 435 participants from Germany (*n* = 135), Spain (*n* = 133), Switzerland (*n* = 20), and the United States (*n* = 147) (Figure 1). They also examined the possibility that HLA‐C*04:01 might have contributed to adverse outcomes via exposing individuals to a more severe inflammatory state, and they observed that these carriers had higher CRP levels (*p* = 0.02).^53^

**TABLE 1:** Description of studies and the results.

HLA allele	Outcome			*p* Value	Study			Reference
	Severity	Susceptibility	Protection		Region	Study sample size	Study design	
HLA‐A*01		✓		***p* = 0.040**	74 countries		Cross‐sectional	[51]
HLA‐A*01:01	✓	✓		***p* = 0.0002**	Russia	539	Cross‐sectional	[55]
HLA‐A*01:01 g		✓			Italy	104,135	Cross‐sectional	[56]
HLA‐A*02			✓	***p* = 0.0536**	UK	10,388	Cross‐sectional	[57]
HLA‐A*02:01			✓	***p* = 0.0146**	Russia	539	Cross‐sectional	[55]
HLA‐A*02:01 g			✓		Italy	104,135	Cross‐sectional (Population‐based)	[56]
HLA‐A*02:05			✓		Sardinia (Italy)	801	Cross‐sectional	[22]
HLA‐A*03	✓	✓		***p*** = 0.047	Spain	3958	Cross‐sectional	[42]
HLA‐A*03:01			✓	***p* = 0.0075**	Russia	539	Cross‐sectional	[55]
HLA‐A*11	✓			***p* = 0.051**	Spain	3958	Cross‐sectional	[42]
HLA‐A*11:01	✓			***p*** = 0.0099	USA	126	Cross‐sectional	[58]
HLA‐A*11:01:01:01	✓			***p*c = 0.013**	Japanese	613	Cross‐sectional	[61]
HLA‐A*23:01	✓			***p*c = 0.038**	Sardinia (Italy)	801	Cross‐sectional	[22]
HLA‐A*26			✓	***p* = 0.0198**	UK	10,388	Cross‐sectional	[57]
HLA‐A*30:02	✓			***p* = 0.0007; *p*c = 0.008**	Sardinia (Italy)	801	Cross‐sectional	[22]
HLA‐A*30:02		✓		***p*_adj = 0.0134**	Midwestern US	22,234	Case–control	[52]
HLA‐A*32			✓	*p* = 0.004	Spain	3958	Cross‐sectional	[42]
HLA‐B*07		✓		***p* = 0.00081**	74 countries		Cross‐sectional	[51]
HLA‐B*08		✓		***p* = 0.047**	74 countries		Cross‐sectional	[51]
HLA‐B*08:01 g		✓			Italy	104,135	Cross‐sectional	[56]
HLA‐B*12			✓	*p* = 0.015	Hong Kong	4376	Cross‐sectional	[62]
HLA‐B*14:02	✓			***p* = 0.0007; *p*c = 0.008**	Sardinia (Italy)	801	Cross‐sectional	[22]
HLA‐B*15			✓		Egypt			[27]
HLA‐B*18:01 g			✓		Italy	104,135	Cross‐sectional	[56]
HLA‐B*27		✓	✓	*p* = 0.047	Hong Kong	4376	Cross‐sectional	[62]
HLA‐B*27:07	✓			***p* = 0.00001**	Italy	1116	Cross‐sectional	[63]
HLA‐B*35			✓	*p* = 0.050	South Asia	95	Cross‐sectional	[32]
HLA‐B*39	✓			*p* = 0.02	Spain	3958	Cross‐sectional	[42]
HLA‐B*41	✓				Egypt			[27]
HLA‐B*42	✓				Egypt			[27]
HLA‐B*44			✓	***p* = 0.0105**	UK	10,388	Cross‐sectional	[57]
HLA‐B*44		✓		***p* = 0.05**	Italy	490,926	Cross‐sectional	[64]
HLA‐B*44		✓		***p* = 0.047**	74 countries		Cross‐sectional	[51]
HLA‐B*51	✓			*p* = 0.027	South Asia	95	Cross‐sectional	[32]
HLA‐B*51:01			✓	*p*bonf = 0.06	Armenia	299	Cross‐sectional	[59]
HLA‐B*52:01:01:02	✓			***p*c = 0.043**	Japan	613	Cross‐sectional	[61]
HLA‐B*58:01	✓			*p* = 0.0131	Italy	1116	Cross‐sectional	[63]
HLA‐B*58:01			✓		Sardinia (Italy)	801	Cross‐sectional	[22]
HLA‐B22		✓		***p* = 0.002 − *p* = 0.032**	Hong Kong	4376	Cross‐sectional	[62]
HLA‐B40		✓		***p* = 0.049**	Sardinia (Italy)	801	Cross‐sectional	[22]
HLA‐C*01	✓			***p* = 0.09**	Spain	3958	Cross‐sectional	[42]
HLA‐C*01		✓		***p* = 0.042**	Italy	490,926	Cross‐sectional	[64]
HLA‐C*04:01		✓		***p* = 0.012**	Sardinia (Italy)	801	Cross‐sectional	[22]
HLA‐C*04:01	✓			***p*bonf = 0.025**	Armenia	299	Cross‐sectional	[59]
HLA‐C*04:01	✓	✓		***p*_unadj = 0.02**	Midwestern US	22,234	Case–control	[52]
HLA‐C*04:01	✓	✓		***p* = 0.0082**	USA	126	Cross‐sectional	[58]
HLA‐C*04:01:01:01	✓			***p* = 0.01 − *p*c = 0.02**	India	96	Observational study	[60]
HLA‐C*05			✓	***p* = 0.0215**	UK	10,388	Cross‐sectional	[57]
HLA‐C*05		✓		***p* = 0.00032 − *p*c = 0.000027**	74 countries		Cross‐sectional	[51]
HLA‐C*06:02			✓	*p* = 0.0053	Italy	1116	Cross‐sectional	[63]
HLA‐C*07			✓		Egypt			[27]
HLA‐C*07:01			✓		Sardinia (Italy)	801	Cross‐sectional	[22]
HLA‐C*07:01 g			✓		Italy	104,135	Cross‐sectional	[56]
HLA‐C*07:01 g		✓			Italy	104,135	Cross‐sectional	[56]
HLA‐C*08:02	✓			***p* = 0.0007; *p*c = 0.008**	Sardinia (Italy)	801	Cross‐sectional	[22]
HLA‐C*12			✓		Egypt			[27]
HLA‐C*12:02:02:01	✓			***p*c = 0.021**	Japan	613	Cross‐sectional	[61]
HLA‐C*16	✓			*p* = 0.02	Spain	3958	Cross‐sectional	[42]
HLA‐C*16	✓				Egypt			[27]
HLAC*17	✓				Egypt			[27]
HLA‐DPA1*01:03:01:02	✓			***p* = 0.0004 − *p*c = 0.01**	India	96	Observational study	[60]
HLA‐DPB1*04:01:01:41	✓			*p* = 0.04 **−** *p*c = 0.38	India	96	Observational study	[60]
HLA‐DQA1*01:01	✓			*p* = 0.007	UK	8661	Cross‐sectional	[18]
HLA‐DQA1*01:02	✓			***p* = 0.0097**	USA	126	Cross‐sectional	[58]
HLA‐DQA1*03:01:01:01	✓			*p* = 0.001	India	96	observational study	[60]
HLA‐DQB1*04	✓			***p* = 0.051**	Spain	3958	Cross‐sectional	[42]
HLA‐DQB1*05:01	✓			*p* = 0.007	UK	8661	Cross‐sectional	[18]
HLA‐DQB1*06		✓		***p* = 0.0523**	UK	10,388	Cross‐sectional	[57]
HLA‐DQB1*06:02	✓			***p* = 0.0001**	Italy	1116	Cross‐sectional	[63]
HLA‐DRB1*01	✓			*p* = 0.02	Mexico	71,099	Cross‐sectional	[65]
HLA‐DRB1*01:01			✓	*p* = 0.007	UK	8661	Cross‐sectional	[18]
HLA‐DRB1*03:01			✓	*p* = 0.001	Sardinia (Italy)	801	Cross‐sectional	[22]
HLA‐DRB1*03:01 g		✓			Italy	104,135	Cross‐sectional	[56]
HLA‐DRB1*04:01			✓	***p* = 0.003**	UK	8661	Cross‐sectional	[18]
HLA‐DRB1*04:02	✓				UK	8661	Cross‐sectional	[18]
HLA‐DRB1*04:05	✓				UK	8661	Cross‐sectional	[18]
HLA‐DRB1*07:01			✓	*p* = 0.0339	Italy	1116	Cross‐sectional	[63]
HLA‐DRB1*08	✓			***p*c = 0.036**	Italy	56,304	Cross‐sectional	[66]
HLA‐DRB1*08:01	✓			***p*c = 0.024**	Sardinia (Italy)	801	Cross‐sectional	[22]
HLA‐DRB1*08:02		✓		***p*_adj = 0.03**	Midwestern US	22,234	Case–control	[52]
HLA‐DRB1*10		✓		***p* = 0.0144**	UK	10,388	Cross‐sectional	[57]
HLA‐DRB1*11	✓			*p* = 0.0185	UK	10,388	Cross‐sectional	[57]
HLA‐DRB1*11:04 g			✓		Italy	104,135	Cross‐sectional	[56]
HLA‐DRB1*13	✓			*p* = 0.049	South Asia	95	Cross‐sectional	[32]
HLA‐DRB1*15		✓			UK	10,388	Cross‐sectional	[57]
HLA‐DRB1*15:01	✓			***p* = 0.0015**	Italy	1116	Cross‐sectional	[63]
HLA‐DRB1*15:01	✓			*p* = 0.001	UK	8661	Cross‐sectional	[18]
HLA‐DRB5*01:01:01:02	✓			***p* = 0.02 − *p*c = 0.01**	India	96	Observational study	[60]

*Note*: The bolded items include the results of alleles that were still statistically significant.

In summary, while there are still limitations and heterogeneity in study designs, the identification of common alleles such as HLA‐A*02 and HLA‐C*04 in various studies can provide insights into the genetic susceptibility and severity of COVID‐19. The allele frequency distribution of these HLA variants varies significantly between areas, suggesting that genetic factors may contribute to the differences in COVID‐19 incidence and severity observed across different regions and populations. Further research is required to fully understand the impact of host genetic variation on COVID‐19 outcomes and to develop personalized treatments and interventions.

## 3 CONCLUSION

The COVID‐19 pandemic has affected millions of people worldwide since its emergence in 2020. The severity and spread of the disease have been attributed to various factors, including genetic variations among different populations. GWASs have provided vital insights into the relationship between host genetic variation and COVID‐19 susceptibility and severity. Analysis of HLA allele frequency distribution revealed significant differences across various populations, which may explain some of the observed differences in COVID‐19 onset and outcomes. However, limitations such as small sample sizes, heterogeneity in study designs, and variable allele frequencies between populations have made it challenging to draw robust conclusions. Despite these limitations, based on the study, the allele with the highest predicted capacity to bind with SARS‐CoV‐2 peptides is HLA‐A*02, which was also found to have a preventive benefit against susceptibility and mortality in some clinical settings. On the other hand, HLA‐C*04 was identified as a potential risk allele and positively correlated with SARS‐CoV‐2 susceptibility and severity, even doubling the risk of intubation when present in only one allele. Therefore, HLA‐A*02 and HLA‐C*04 can be suggested as the alleles with the lowest and highest risk of contracting COVID‐19, respectively. These findings are crucial for understanding the genetic basis of COVID‐19 and can guide future research in developing targeted prevention and treatment strategies. Further studies with larger sample sizes and globally diverse populations are required to overcome the limitations and validate the findings of previous GWAS on COVID‐19.

## CONFLICT OF INTEREST STATEMENT

The authors declare that there are no conflicts of interest.

## Data Availability

Data sharing is not applicable to this article as no new data were created or analyzed in this study.

## References

[1] Crux NB , Elahi S . Human leukocyte antigen (HLA) and immune regulation: how do classical and non‐classical HLA alleles modulate immune response to human immunodeficiency virus and hepatitis C virus infections? Front Immunol. 2017;8:832. 28769934 10.3389/fimmu.2017.00832 PMC5513977

[2] Bodmer W . The HLA system: structure and function. J Clin Pathol. 1987;40(9):948‐958. 3312304 10.1136/jcp.40.9.948 PMC1141164

[3] Liu B , Shao Y , Fu R . Current research status of HLA in immune‐related diseases. Immun Inflamm Dis. 2021;9(2):340‐350. 33657268 10.1002/iid3.416 PMC8127548

[4] SeyedAlinaghi S , Karimi A , Barzegary A , et al. COVID‐19 mortality in patients with immunodeficiency and its predictors: a systematic review. Eur J Med Res. 2022;27(1):195. 36209202 10.1186/s40001-022-00824-7 PMC9547631

[5] Ryschich E , Nötzel T , Hinz U , et al. Control of T‐cell–mediated immune response by HLA class I in human pancreatic carcinoma. Clin Cancer Res. 2005;11(2):498‐504. 15701833

[6] SeyedAlinaghi S , Mirzapour P , Pashaei Z , et al. The impacts of COVID‐19 pandemic on service delivery and treatment outcomes in people living with HIV: a systematic review. AIDS Res Ther. 2023;20(1):4. 36609313 10.1186/s12981-022-00496-7 PMC9821373

[7] Schott G , Garcia‐Blanco MA . MHC class III RNA binding proteins and immunity. RNA Biol. 2021;18(5):640‐646. 10.1080/15476286.2020.1860388 PMC8163431 33280511

[8] Hamidi‐Sofiani V , Rakhshi R , Moradi N , Zeynali P , Nakhaie M , Behboudi E . Oncolytic viruses and pancreatic cancer. Cancer Treat Res Commun. 2022;31:100563. 35460973 10.1016/j.ctarc.2022.100563

[9] Zandi M , Soltani S , Tabibzadeh A , et al. Partial sequence conservation of SARS‐CoV‐2 NSP‐2, NSP‐12, and spike in stool samples from Abadan, Iran. Biotechnol Appl Biochem. 2023;70(1):201‐209. 35396867 10.1002/bab.2343 PMC9082511

[10] Faraji SN , Raee MJ , Hashemi SMA , et al. Human interaction targets of SARS‐CoV‐2 spike protein: a systematic review. Eur J Inflamm. 2022;20:1721727X221095382.

[11] Amir H . Strategies in preventing the transmission of COVID‐19 a quarantine, isolation, lockdown, tracing, testing and treatment (3t): literature review. Asia Pac J Health Manag. 2022;17(2):1465.

[12] Ahmad A , Hussaan M , Batool F , et al. Social distancing and quarantine as COVID‐19 control remedy. In: Ahmedah HT , Riaz M , Ahmed S , Moga MA , eds. The COVID‐19 Pandemic. Apple Academic Press; 2022:145‐178.

[13] Chathappady House NN , Palissery S , Sebastian H . Corona viruses: a review on SARS, MERS and COVID‐19. Microbiol Insights. 2021;14:11786361211002481. 33795938 10.1177/11786361211002481 PMC7983408

[14] Emadi MS , Soltani S , Noori B , et al. Highly conserve sequences in envelope, nucleoprotein and RNA‐dependent RNA polymerase of SARS‐CoV‐2 in nasopharyngeal samples of the COVID‐19 patients; a diagnostic target for further studies. J Cell Mol Anesth. 2022;7(2):78‐83.

[15] Chowdhury MA , Hossain N , Kashem MA , Shahid MA , Alam A . Immune response in COVID‐19: a review. J Infect Public Health. 2020;13(11):1619‐1629. 32718895 10.1016/j.jiph.2020.07.001 PMC7359800

[16] Migliorini F , Torsiello E , Spiezia F , Oliva F , Tingart M , Maffulli N . Association between HLA genotypes and COVID‐19 susceptibility, severity and progression: a comprehensive review of the literature. Eur J Med Res. 2021;26(1):1‐9. 34344463 10.1186/s40001-021-00563-1 PMC8329616

[17] Hammer C , Begemann M , McLaren PJ , et al. Amino acid variation in HLA class II proteins is a major determinant of humoral response to common viruses. Am J Hum Genet. 2015;97(5):738‐743. 26456283 10.1016/j.ajhg.2015.09.008 PMC4667104

[18] Langton DJ , Bourke SC , Lie BA , et al. The influence of HLA genotype on the severity of COVID‐19 infection. HLA. 2021;98(1):14‐22. 33896121 10.1111/tan.14284 PMC8251294

[19] Tomita Y , Ikeda T , Sato R , Sakagami T . Association between HLA gene polymorphisms and mortality of COVID‐19: an in silico analysis. Immun Inflamm Dis. 2020;8(4):684‐694. 33047883 10.1002/iid3.358 PMC7654404

[20] Wang W , Zhang W , Zhang J , He J , Zhu F . Distribution of HLA allele frequencies in 82 Chinese individuals with coronavirus disease‐2019 (COVID‐19). HLA. 2020;96(2):194‐196. 32424945 10.1111/tan.13941 PMC7276866

[21] Kakodkar P , Dokouhaki P , Wu F , et al. The role of the HLA allelic repertoire on the clinical severity of COVID‐19 in Canadians, living in the Saskatchewan province. Hum Immunol. 2023;84(3):163‐171. 36707385 10.1016/j.humimm.2023.01.003 PMC9852320

[22] Roberto L , Campagna M , Silvia D , et al. Human leukocyte antigen complex and other Immunogenetic and clinical factors influence susceptibility or protection to SARS‐CoV‐2 infection and severity of the disease course. The Sardinian experience. Front Immunol. 2020;11:605688. 33343579 10.3389/fimmu.2020.605688 PMC7746644

[23] Gupta R , Misra A . COVID19 in south Asians/Asian Indians: heterogeneity of data and implications for pathophysiology and research. Diabetes Res Clin Pract. 2020;165:108267. doi:10.1016/j.diabres.2020.108267 32533988 PMC7832204

[24] Oyarzun P , Ellis JJ , Gonzalez‐Galarza FF , et al. A bioinformatics tool for epitope‐based vaccine design that accounts for human ethnic diversity: application to emerging infectious diseases. Vaccine. 2015;33(10):1267‐1273. 25629524 10.1016/j.vaccine.2015.01.040

[25] Ahammad I , Lira SS . Designing a novel mRNA vaccine against SARS‐CoV‐2: an immunoinformatics approach. Int J Biol Macromol. 2020;162:820‐837. 32599237 10.1016/j.ijbiomac.2020.06.213 PMC7319648

[26] Barquera R , Collen E , Di D , et al. Binding affinities of 438 HLA proteins to complete proteomes of seven pandemic viruses and distributions of strongest and weakest HLA peptide binders in populations worldwide. HLA. 2020;96(3):277‐298. doi:10.1111/tan.13956 32475052 PMC7300650

[27] Abdelhafiz AS , Ali A , Fouda MA , et al. HLA‐B*15 predicts survival in Egyptian patients with COVID‐19. Hum Immunol. 2022;83(1):10‐16. doi:10.1016/j.humimm.2021.09.007 34607724 PMC8485223

[28] Deb P , Zannat KE , Talukder S , Bhuiyan AH , Jilani MS , Saif‐Ur‐Rahman KM . Association of HLA gene polymorphism with susceptibility, severity, and mortality of COVID‐19: a systematic review. HLA. 2022;99(4):281‐312. 35067002 10.1111/tan.14560

[29] Fakhkhari M , Caidi H , Sadki K . HLA alleles associated with COVID‐19 susceptibility and severity in different populations: a systematic review. Egypt J Med Hum Genet. 2023;24(1):10. 36710951 10.1186/s43042-023-00390-5 PMC9867832

[30] Bordon Y . Asymptomatic SARS‐CoV‐2 infections linked to HLA‐B* 15: 01. Nat Rev Genet. 2023;24(10):663. 10.1038/s41576-023-00641-6 37507492

[31] Tavasolian F , Rashidi M , Hatam GR , et al. HLA, immune response, and susceptibility to COVID‐19. Front Immunol. 2020;11:601886. doi:10.3389/fimmu.2020.601886 33488597 PMC7820778

[32] Naemi FMA , Al‐Adwani S , Al‐Khatabi H , Al‐Nazawi A . Association between the HLA genotype and the severity of COVID‐19 infection among south Asians. J Med Virol. 2021;93(7):4430‐4437. doi:10.1002/jmv.27003 33830530 PMC8251353

[33] Dobrijević Z , Gligorijević N , Šunderić M , et al. The association of human leucocyte antigen (HLA) alleles with COVID‐19 severity: a systematic review and meta‐analysis. Rev Med Virol. 2023;33(1):e2378. 35818892 10.1002/rmv.2378 PMC9349710

[34] Augusto DG , Murdolo LD , Chatzileontiadou DS , et al. A common allele of HLA is associated with asymptomatic SARS‐CoV‐2 infection. Nature. 2023;620(7972):128‐136. 37468623 10.1038/s41586-023-06331-x PMC10396966

[35] Ng MH , Lau KM , Li L , et al. Association of human‐leukocyte‐antigen class I (B*0703) and class II (DRB1*0301) genotypes with susceptibility and resistance to the development of severe acute respiratory syndrome. J Infect Dis. 2004;190(3):515‐518. doi:10.1086/421523 15243926 PMC7109646

[36] Nguyen A , David JK , Maden SK , et al. Human leukocyte antigen susceptibility map for severe acute respiratory syndrome coronavirus 2. J Virol. 2020;94(13):e00510‐20. 32303592 10.1128/JVI.00510-20 PMC7307149

[37] Gálvez J , Gálvez JJ , García‐Peñarrubia P . Is TCR/pMHC affinity a good estimate of the T‐cell response? An answer based on predictions from 12 phenotypic models. Front Immunol. 2019;10:349. 30886616 10.3389/fimmu.2019.00349 PMC6410681

[38] Yung YL , Cheng CK , Chan HY , et al. Association of HLA‐B22 serotype with SARS‐CoV‐2 susceptibility in Hong Kong Chinese patients. HLA. 2021;97(2):127‐132. 33179437 10.1111/tan.14135 PMC7898481

[39] Augusto DG , Hollenbach JA . HLA variation and antigen presentation in COVID‐19 and SARS‐CoV‐2 infection. Curr Opin Immunol. 2022;76:102178. 35462277 10.1016/j.coi.2022.102178 PMC8947957

[40] Correale P , Mutti L , Pentimalli F , et al. HLA‐B* 44 and C* 01 prevalence correlates with COVID‐19 spreading across Italy. Int J Mol Sci. 2020;21(15):5205. 32717807 10.3390/ijms21155205 PMC7432860

[41] Ebrahimi S , Ghasemi‐Basir HR , Majzoobi MM , Rasouli‐Saravani A , Hajilooi M , Solgi G . HLA‐DRB1*04 may predict the severity of disease in a group of Iranian COVID‐19 patients. Hum Immunol. 2021;82(10):719‐725. doi:10.1016/j.humimm.2021.07.004 34294460 PMC8275473

[42] Lorente L , Martín MM , Franco A , et al. HLA genetic polymorphisms and prognosis of patients with COVID‐19. Med Intensiva. 2021;45(2):96‐103. doi:10.1016/j.medin.2020.08.004 38620408 PMC7474921

[43] Zandi M , Behboudi E , Shojaei MR , et al. Letter to the Editor Regarding “An overview on serology and molecular tests for COVID‐19: an important challenge of the current century (doi: 10.22034/iji.2021.88660.1894)”. Iran J Immunol. 2022;19(3):337. 36190387 10.22034/iji.2022.91791.2107

[44] Pareek M , Bangash MN , Pareek N , et al. Ethnicity and COVID‐19: an urgent public health research priority. Lancet. 2020;395(10234):1421‐1422. doi:10.1016/s0140-6736(20)30922-3 32330427 PMC7173801

[45] Ellinghaus D , Degenhardt F , Bujanda L , et al. Genomewide association study of severe Covid‐19 with respiratory failure. N Engl J Med. 2020;383(16):1522‐1534. doi:10.1056/NEJMoa2020283 32558485 PMC7315890

[46] Horowitz JE , Kosmicki JA , Damask A , et al. Genome‐wide analysis in 756,646 individuals provides first genetic evidence that ACE2 expression influences COVID‐19 risk and yields genetic risk scores predictive of severe disease. medRxiv. 2021. doi:10.1101/2020.12.14.20248176

[47] Pairo‐Castineira E , Clohisey S , Klaric L , et al. Genetic mechanisms of critical illness in COVID‐19. Nature. 2021;591(7848):92‐98. doi:10.1038/s41586-020-03065-y 33307546

[48] Mapping the human genetic architecture of COVID‐19. Nature. 2021;600(7889):472‐477. doi:10.1038/s41586-021-03767-x 34237774 PMC8674144

[49] Shelton JF , Shastri AJ , Ye C , et al. Trans‐ancestry analysis reveals genetic and nongenetic associations with COVID‐19 susceptibility and severity. Nat Genet. 2021;53(6):801‐808. doi:10.1038/s41588-021-00854-7 33888907

[50] Ben Shachar S , Barda N , Manor S , et al. MHC Haplotyping of SARS‐CoV‐2 patients: HLA subtypes are not associated with the presence and severity of COVID‐19 in the Israeli population. J Clin Immunol. 2021;41(6):1154‐1161. doi:10.1007/s10875-021-01071-x 34050837 PMC8164405

[51] Sakuraba A , Haider H , Sato T . Population difference in allele frequency of HLA‐C*05 and its correlation with COVID‐19 mortality. Viruses. 2020;12(11):1333. doi:10.3390/v12111333 33233780 PMC7699862

[52] Schindler E , Dribus M , Duffy BF , et al. HLA genetic polymorphism in patients with coronavirus disease 2019 in Midwestern United States. HLA. 2021;98(4):370‐379. doi:10.1111/tan.14387 34338446 PMC8429120

[53] Weiner J , Suwalski P , Holtgrewe M , et al. Increased risk of severe clinical course of COVID‐19 in carriers of HLA‐C*04:01. EClinicalMedicine. 2021;40:101099. doi:10.1016/j.eclinm.2021.101099 34490415 PMC8410317

[54] Iturrieta‐Zuazo I , Rita CG , García‐Soidán A , et al. Possible role of HLA class‐I genotype in SARS‐CoV‐2 infection and progression: a pilot study in a cohort of COVID‐19 Spanish patients. Clin Immunol. 2020;219:108572. doi:10.1016/j.clim.2020.108572 32810602 PMC7428760

[55] Shkurnikov M , Nersisyan S , Jankevic T , et al. Association of HLA class I genotypes with severity of coronavirus Disease‐19. Front Immunol. 2021;12:641900. doi:10.3389/fimmu.2021.641900 33732261 PMC7959787

[56] Pisanti S , Deelen J , Gallina AM , et al. Correlation of the two most frequent HLA haplotypes in the Italian population to the differential regional incidence of COVID‐19. J Transl Med. 2020;18(1):352. doi:10.1186/s12967-020-02515-5 32933522 PMC7491019

[57] Poulton K , Wright P , Hughes P , et al. A role for human leucocyte antigens in the susceptibility to SARS‐Cov‐2 infection observed in transplant patients. Int J Immunogenet. 2020;47(4):324‐328. doi:10.1111/iji.12505 32623831 PMC7361549

[58] Warren RL , Birol I . Retrospective in silico HLA predictions from COVID‐19 patients reveal alleles associated with disease prognosis. medRxiv. 2020. doi:10.1101/2020.10.27.20220863

[59] Hovhannisyan A , Madelian V , Avagyan S , et al. HLA‐C*04:01 affects HLA class I heterozygosity and predicted affinity to SARS‐CoV‐2 peptides, and in combination with age and sex of Armenian patients contributes to COVID‐19 severity. Front Immunol. 2022;13:769900. doi:10.3389/fimmu.2022.769900 35185875 PMC8850920

[60] Vishnubhotla R , Sasikala M , Ketavarapu V , Reddy DN . High‐resolution HLA genotyping identifies alleles associated with severe COVID‐19: a preliminary study from India. Immun Inflamm Dis. 2021;9(4):1781‐1785. doi:10.1002/iid3.481 34289534 PMC8426675

[61] Khor S‐S , Omae Y , Nishida N , et al. HLA‐A*11:01:01:01, HLA‐C*12:02:02:01‐HLA‐B*52:01:02:02, age and sex are associated with severity of Japanese COVID‐19 with respiratory failure. Front Immunol. 2021;12:658570. doi:10.3389/fimmu.2021.658570 33968060 PMC8100314

[62] Yung YL , Cheng CK , Chan HY , et al. Association of HLA‐B22 serotype with SARS‐CoV‐2 susceptibility in Hong Kong Chinese patients. HLA. 2021;97(2):127‐132. 33179437 10.1111/tan.14135 PMC7898481

[63] Novelli A , Andreani M , Biancolella M , et al. HLA allele frequencies and susceptibility to COVID‐19 in a group of 99 Italian patients. HLA. 2020;96(5):610‐614. 32827207 10.1111/tan.14047 PMC7461491

[64] Correale P , Mutti L , Pentimalli F , et al. HLA‐B*44 and C*01 Prevalence Correlates with Covid19 Spreading across Italy. Int J Mol Sci. 2020;21(15):5205. 32717807 10.3390/ijms21155205 PMC7432860

[65] Romero‐López JP , Carnalla‐Cortés M , Pacheco‐Olvera DL , et al. A bioinformatic prediction of antigen presentation from SARS‐CoV‐2 spike protein revealed a theoretical correlation of HLA‐DRB1*01 with COVID‐19 fatality in Mexican population: an ecological approach. J Med Virol. 2021;93(4):2029‐2038. 32986250 10.1002/jmv.26561 PMC7537233

[66] Amoroso A , Magistroni P , Vespasiano F , et al. HLA and AB0 polymorphisms may influence SARS‐CoV‐2 infection and COVID‐19 severity. Transplantation. 2021;105(1):193‐200. 33141807 10.1097/TP.0000000000003507

